# Effects of NaCl on Antioxidant, Antifungal, and Antibacterial Activities in Safflower Essential Oils

**DOI:** 10.3390/plants10122809

**Published:** 2021-12-18

**Authors:** Houneida Attia, Jamel Harrathi, Khalid H. Alamer, Fatin A. Alsalmi, Christian Magné, Maha Khalil

**Affiliations:** 1 Department of Biology, College of Sciences, Taif University, P.O. Box 11099, Taif 21944, Saudi Arabia; alsalmi.f@tu.edu.sa (F.A.A.); maha.ak@tu.edu.sa (M.K.); 2 Unité de Physiologie et Biochimie De La Réponse Des Plantes Aux Contraintes Abiotiques, Département de Biologie, FST, Université Tunis El Manar, Tunis 1068, Tunisia; harrathi.jamel@yahoo.fr; 3 Department of Biology, Science and Arts College, Rabigh Campus, King Abdulaziz University, Jeddah 21589, Saudi Arabia; kalamer@kau.edu.sa; 4 Géoarchitecture Territoires, Urbanisation, Biodiversité, Environnement, Université de Brest, EA 7462, CS 93837, CEDEX 3, F-29238 Brest, France; Christian.Magne@univ-brest.fr

**Keywords:** antimicrobial activity, antioxidant activity, essential oils, salt, safflower

## Abstract

The present study aims to evaluate the antioxidant and antimicrobial activity of essential oils (EO) extracted from safflower plants grown in the absence and presence of NaCl, 50 mM. Plants treated with 50 mM of NaCl showed decreases in root, stem, and leaf dry weight. Results of the essential oils showed that roots have a higher EO yield than leaves and stems. Salinity caused a decrease in this yield in roots and leaves but not in stems. The compounds identified in the EO extracted from these organs belong to seven chemical classes of which the dominant class is the sesquiterpene hydrocarbons. The chemotype of *C. tinctorius* EO is variable depending on the organ and the treatment. The safflower essential oils showed low antioxidant, antiradical, and iron-reducing activities compared to those of the positive control (BHT). In an antifungal activity test, only two strains, *Aspergillus niger* and *Candida albicans*, were found to be highly sensitive to these oils as they showed almost total inhibition of their growth. For antibacterial activity, safflower EOs showed significant antimicrobial activity against *Bacillus subtilis*, *Bacillus cereus*, and *Xanthomonas campestris* in both control and NaCl-treated plants: for these three strains, total inhibition of growth was noted at 50,000 ppm of EO in leaves and roots; whereas for stems, total inhibition was noted only for the third strain (*Xanthomonas campestris*). For other strains, this inhibition was variable and weak. Salt was found to have no effect on the activities of safflower EOs.

## 1. Introduction

Safflower, *Carthamus tinctorius* L., is a polyvalent crop widely used in pharmacology and medicine for its seeds, flowers, and foliage, which have many biological properties and functional uses related to food [1]. Safflower oil, rich in linolenic acid, is, for example, well-established as a means of lowering blood cholesterol levels [2] and is also clinically used for treating diseases such as catalase, osteoporosis, and rheumatoid arthritis [3]. The protective effects of its seeds in preventing fractures and the loss of bone were also documented [4]. It is cultivated in the Mediterranean basin as a dye plant for the coloring power of its flowers, in Asia and Latin America as an oilseed. In Chinese traditional medicine, its flowers have been applied to treat cardiovascular and brain disorders [5]. More recently, Alkhafaji et al. [6] reported that extracts of its flowers have numerous antioxidant, antimicrobial, anti-inflammatory, antidepressant, and antitumor properties.

Salt is a critical abiotic constraint, which can decrease agricultural yield. The area expansion under salinity has expanded considerably. Depending on the gravity and durability of the salt stress, plants exhibit different responses. First, salinity restricts plant growth because of the osmotic material present at the roots. In addition, salinity induces hypertonic stress on plants [7]. Under salinity stress, ions, mainly Na^+^ and Cl^−^, accumulate in plant tissues and lead to important physiological and biochemical perturbations, the production of reactive oxygen species (ROS), and the inhibition of K^+^ absorption [7]. Consequently, these events lead to reduced plant growth and development. In the previous survey, the plant growth properties, essential oil (EO) levels, and quality of Mentha reduced under salt stress [8].

Throughout the world, safflower is cultivated for the production of oil because of its seeds’ richness in linoleic acid. This plant is propagated in several regions due to its importance. Generally, in arid and semi-arid regions, water loaded with salt is used for this crop. Moreover, it is known that the production and quality of essential oils depend on climatic, edaphic, and genetic conditions; and the age and stage of development of the plant [9]. The study of the effects of salinity on the physiology of safflower (*Carthamus tinctorius* L.) is useful in view of its medicinal, pharmaceutical, and agri-food interests. Safflower is considered a moderately salinity-tolerant plant [10]. It is an oilseed crop suitable for cultivation in arid agricultural areas based on its high tolerance to both heat and cold, and also can be cultivated in irrigated agricultural zones because of its tolerance to salinity and weeds [11]. Nevertheless, Bassil and Kaffka [12] showed that salinity induces a decrease in biomass, plant height, number of flower heads per plant, leaf area, and the number of days to maturity.

Nowadays, the scientific interest of this species is principally due to the high quality of its vegetable oil for feeding and commercial applications [13]. The nutritive value of safflower oil is, in fact, the same as that of olive oil [14] and, for this reason, the species has acquired importance in recent years due to human consumption in arid and semi-arid regions. Traditional safflower oil, which is rich in polyunsaturated linoleic acid, is appreciated for human health benefits because its high linoleic content induces significant decreases in the level of blood cholesterol [15], with the exception that it is not appropriate for prolonged deep-frying because of its low oxidative stability at elevated temperatures. Contrarily, oil rich in monounsaturated oleic acid has high oxidative stability, making it appropriate for alimentary purposes and an appropriate alternative to olive oil in arid and semi-arid regions of the world [14].

Essential oils have the ability to inhibit the reproduction of bacteria and fungi and destroy them [16]. According to the literature, terpenoids have significant antimicrobial activity [17] Recently, many studies have focused on determining the antibacterial activity of EOs from a large number of aromatic and medicinal plants (AMPs) [18]. Allen and Thomas [19] showed that safflower contains a compound, Trans-trans-3,11-tridecadiene-5,7,9-triyne-1,2-diol, with antifungal properties. Khémiri et al. [20] revealed that safflower (*Carthamus tinctorius* L.) seed oil displayed high antioxidant and antimicrobial effects. Furthermore, extracts of *Carthamus tinctorius* L. flowers harvested at the last stage of flowering were shown to have significant antimicrobial effects against a fungal strain (*Candida albicans*) and certain bacterial strains (*Escherichia coli*, *Staphylococcus aureus*, *Pseudomonas aeruginosa*, and *Bacillus cereus*). The most intense activity was against *Escherichia coli* with a zone of inhibition of 26 mm [21].

The effect of salinity on EOs depends on the degree of salinity of the environment and the degree of salt tolerance of the species. Indeed, treatment of sunflower with 50 mM NaCl reduces its EO yield [10]. Sarmoum et al. [22] showed that salinity influences the yield of EO from *Rosmarinus officinalis* L. Tounekti et al. [23] showed that the 1,8-cineole content reduced up to 50% by elevation of NaCl concentrations (25 to 200 mM) in *R. officinalis*. On the contrary, Khalid and da Silva [24] demonstrated that the plant of *Calendula officinalis* L., which was subjected to various rates of salt-rich irrigation water (0.39 to 9.38 dS m^−1^) composed of NaCl, CaCl_2_, and MgCl_2_ salts enhanced the EO content and its main constituents (α-cadinol, γ-, and δ-cadinene).

The presented study aimed to determine the impact of NaCl on the growth and essential oils of *C. tinctorius*, as well as the antimicrobial and antioxidant activities (total antioxidant activity, activity against DPPH radical, β-carotene bleaching test, and iron reducing capacity) of essential oils extracted from safflower plants, at the vegetative stage, after 25 days of culture in the absence or presence of 50 mM NaCl.

## 2. Materials and Methods

### 2.1. Plant Material and Growth Conditions

Safflower (*Carthamus tinctorius* L.) surface was sterilized by immersion in 5% (*v*/*v*) calcium hypochlorite with shaking for 15 min, imbibed for 1 day in distilled water, and then sown in Petri dishes with wet filter paper for germination in the dark in a growth room at 25 °C. Six-day-old seedlings were transferred into pots containing 5 L of a quarter diluted nutrient solution [25], for 25 days, then NaCl, 50 mM was either added or not added. The photoperiod was 16 h with 150 Lmol m^−^^2^ s^−^^1^ PAR at the plant level. Day/night temperature and relative humidity regimes were 22/18 °C and 60/80%, respectively. The nutrient solution was continuously aerated.

After 25 days of treatment, plants were harvested and dried at room temperature for measurements of growth (dry weight), and nutrition (Na^+^ and K^+^ content of the different organs)—eight replicates per treatment—and essential oils were extracted by hydrodistillation technique—four replicates per treatment. Extracts were then diluted in methanol for the determination of antioxidant, antifungal, and antibacterial activities. Each parameter was studied in four replicates.

### 2.2. Extraction of Essential Oils

A quantity of 50 g of plant material was placed in a one-liter flask containing 500 mL of water and heated with a balloon heater. The volatile compounds were entrained and condensed by the steam at the level of a refrigerator and recovered in an erlen.

### 2.3. Analysis and Identification of Safflower Essential Oils Constituents

Chromatographic analyses were performed using an Agilent HP μ 6890 gas chromatograph equipped with an HP-Innowax capillary column (30 mm x 0.25 mm x 0.25 μm) and an FID detector (temperature: 250 °C). The carrier gas used is nitrogen (U grade) at a flow rate of 1.6 mL/min. The temperature of the oven was raised to 35 °C for 10 min then it evolves between 35 and 205 °C at a rate of 3 °C/min. Finally, it was kept isothermal at 205 °C for 10 min. A volume of 0.7 μL of solution was injected by the Split mode with a 60:1 ratio. The apparatus is connected to a computer system managing a database allowing the identification of volatile compounds.

The identification of volatile compounds was done by three methods which are: calculation of the Kovàts index (KI), co-chromatography, and gas phase/mass spectrometry coupling.

### 2.4. Antioxidant Activities of Essential Oil

For the evaluation of the free radical scavenging capacity, the DPPH (1,1-diphenyl-2-picrylhydrazyl) assay was used [26]. Briefly, 50 µL aliquots of various concentrations: 5, 10, 25, and 50 mg/L of the oil samples were added to 5 mL of a 0.004% methanol solution of DPPH freshly prepared (the fresh working solution of DPPH was prepared by dissolving 3 mg powder of DPPH reagent into 100 mL of methanol (0.004%). The working solution was kept in a brown bottle in dark conditions until use). After a 30-min incubation period at room temperature, absorbance was read against a blank at 517 nm in Jasco V-630 spectrophotometer (Tokyo, Japan). Decreasing absorbance of DPPH solution indicates an increase in DPPH radical scavenging activity. This activity was given as percent DPPH radical scavenging, which was calculated with the equation:

%DPPH radical scavenging = [(control absorbance–sample absorbance)/control absorbance] × 100.

A β-carotene bleaching assay was carried out according to the method of Kulisic et al. [27] with some modifications. A stock solution of β-carotene-linoleic acid mixture was prepared by dissolving 10 mg β-carotene, 200 mg linoleic acid, and 1 g Tween 40 in 100 mL of chloroform (HPLC grade). The chloroform was removed under vacuum in a rotary evaporator at 50 °C. Then, 50 mL of distilled water saturated with oxygen (30 min, 100 mL/min) was added and the mixture shaken. A 5 mL of this reaction mixture was dispersed to test tubes containing 200 µL of essential oil at different concentrations (5, 10, 25, and 50 mg/L), and the absorbance as t = 0 measured at 490 nm against a blank, consisting of an emulsion without β-carotene. Then the emulsion was incubated for 50 min at room temperature and the absorbance was recorded. The BHT was used as positive control.

The method used for total antioxidant activity was that of Prieto et al. [28]. The antioxidant compounds reduce molybdenum VI to molybdenum V and then, at acidic pH and in presence of phosphate, a green phosphate/molybdenum V complex was formed. This complex was measured by reading the optical density at 695 nm. The reagent consists of 0.6 M sulfuric acid, 28 mM sodium phosphate (NaH_2_PO_4_), and 4 mM ammonium molybdate. Ascorbic acid (Asc) concentrations between 0 and 500 µg/mL were prepared to establish a calibration line. A volume of essential oil diluted in methanol to give concentrations ranging from 5000 to 50,000 ppm was added to 100 µL of diluted extract or diluted ascorbic acid. After homogenization, tubes were incubated for 90 min at 95 °C in a water bath. Optical density was measured at 695 nm and results were expressed as mg ascorbic acid equivalent per g dry matter (mg EAA·g^−1^ DM).

The method used for reducing power was that of Oyaizu [29]. Potassium ferricyanide (K_3_Fe(CN)_6_) provides Fe^3+^ ions, which were reduced to Fe^2+^ depending on the ability of the extract and its antioxidant compounds to give up electrons or not. This method assesses the reducing activity of the extract under test. The increase in absorbance at 700 nm indicates a higher reducing power of the extract.

To obtain concentrations ranging from 5000 to 50,000 ppm, 0.5 mL of phosphate buffer (0.2 M, pH 6.6) and 0.5 mL of 1% K_3_Fe(CN)_6_ were added to one volume of essential oil diluted in methanol. The resulting mixture was incubated for 25 min in an oven at 50 °C. Then 0.5 mL of 10% trichloroacetic acid (TCA) was added to stop the reaction. Centrifugation (650× *g*, 10 min) at room temperature was performed. To 0.5 mL of the supernatant was added 0.5 mL of distilled water and 0.1 mL of 0.1% iron III chloride (FeCl_3_).

The absorbance reading was taken at 700 nm. Results were expressed as 50% effective concentration (EC_50_, µg·mL^−1^). EC_50_ was the concentration corresponding to half the maximum optical density, obtained with high values of sample concentration. It was deduced graphically from the curve expressing the relationship between optical density and concentration.

### 2.5. Antifungal Activities of Essential Oil

The use of a colored indicator, resazurin, as a growth indicator, has already been reported in the literature during antimicrobial activity tests [30,31].

To develop a colored antifungal activity test, we first tried to determine the minimum quantity of resazurin to deposit in each well in order to observe the clearest possible color change. To do this, we tested different concentrations starting with an initial solution of resazurin at 20 mg/mL. It was with 5 µL of dye per well (i.e., 1 mg/mL) that we obtained the most distinct color changes at t = 96 h (final duration).

For the preparation of the plates containing the safflower essential oils, the same protocol was followed as for the antimicrobial activity, except that the positive control was carried out with only the fungal suspension and the negative control was carried out by adding 10 µL of a mixture of antibiotics (Amphotericin B at 2.5 mg·mL^−1^ and Pimaricin at 1 mg·mL^−1^). Once the deposition is complete, the microplates were covered with sterile film in a fume hood and incubated in an oven at 25 °C.

The fungi used in the antifungal tests were: *Aspergillus niger* (ATCC 9029); *Candida albicans* (yeast) (ATCC 10231); *Penicillium frequentans* (ATCC 96048); *Fusarium roseum* (ATCC 28114); *Mucor plumbeus* (ATCC 4740), and *Rhizopus stolonifer* (ATCC 24862).

### 2.6. Antibacterial Activities of Essential Oil

Antimicrobial activity tests were carried out on 96-well microplates (Nunc, Fisher Bioblok). Essential oils were diluted in methanol to obtain concentrations ranging from 50,000 to 5000 ppm. Two hundred µL of these dilutions were deposited in the wells with six repetitions for each deposit. After evaporation of the solvent (methanol) in wells, 100 µL of bacterial suspension at a concentration of 102 bacteria/mL was added under microbiological hot water. A positive control was performed with only suspension of microorganisms, and a negative control was performed by adding 10 µL of antibiotic (5 mg·mL^−1^ streptomycin and 10 mg·mL^−1^ penicillin G) to 100 µL of suspension of microorganisms. Incubation was done at 30 °C for 24 h under sterile film.

After shaking the plates, absorbance was read at 405 nm in a Trertek Multiskan MCC microplate reader. Results of the absorbance reading were calculated as the percentage of growth inhibition of microorganism (relative to the positive control) according to the formula:

% Growth = (AS − AC+)/(AC− − AC+) × 100

AS: Absorbance of the sample

AC: Absorbance of control

The bacterial strains used were: Pseudomonas aeruginosa (ATCC 27853), Pseudomonas fluorescens (ATCC 13525), Bacillus subtilis (ATCC 6633), Listeria monocytogenes (ATCC 35152), Micrococcus luteus (ATCC 10240), Salmonella enterica (ATCC 13314), Escherichia coli (Bacilli Gram^−^, ATCC 1053), Bacillus cereus (Bacilli Gram+, ATCC 6464), Staphylococcus aureus (Cocci Gram^+^, ATCC 33862), and Xanthomonas campestris (ATCC 33913).

### 2.7. Statistical Analysis

Statistical analysis was performed with StatisticaTM software, using two-way analysis of variance (ANOVA) and the Newman–Keuls test for post hoc mean comparison at the significance level of 0.05.

## 3. Results

### 3.1. Plants Morphological Aspect, Growth and Nutritional Status

**Figure 1:**
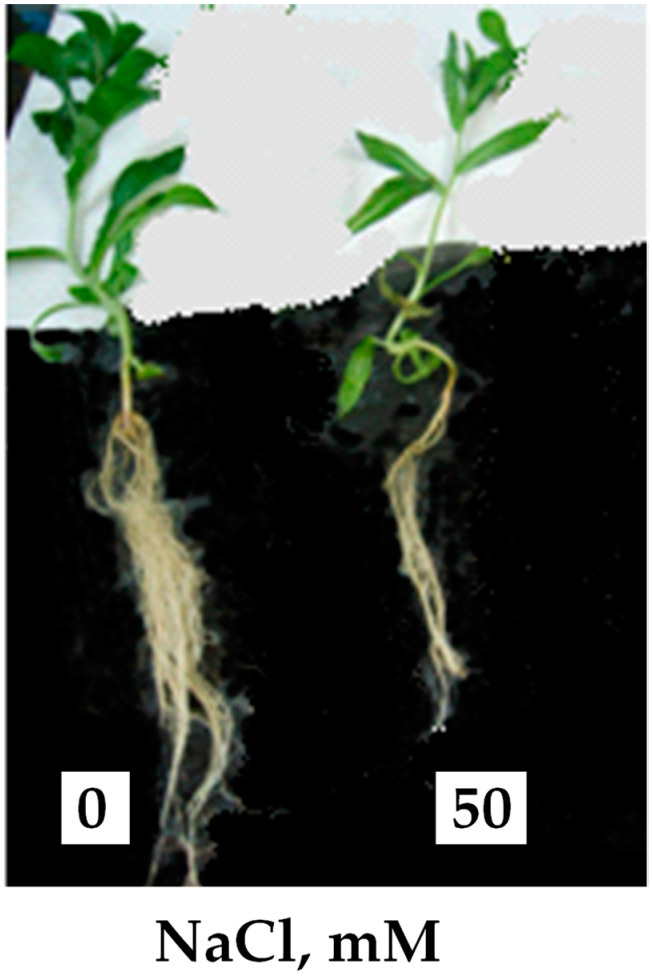
Morphological aspect of *C. tinctorius* plants at the vegetative stage, in the absence and presence of NaCl (50 mM), after 25 days of treatment. Plants were aged 40 days.

The presence of 50 mM NaCl in the culture medium, after 25 days of treatment, leads to morphological changes manifested by a reduction in root length and stem thickness and by a yellowing of the leaves, which first affects the basal leaves and then reaches the youngest ones (Figure 1).

**Figure 2:**
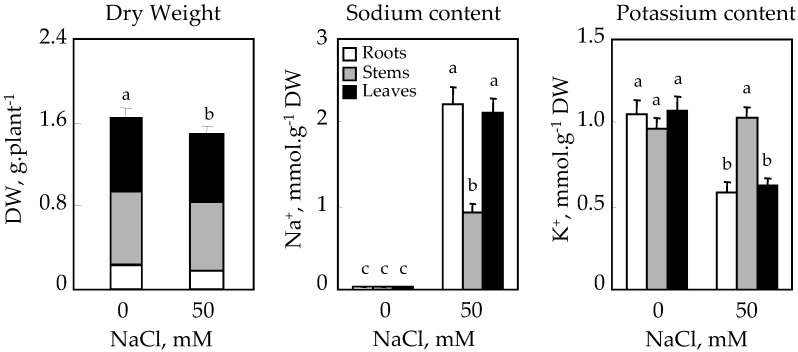
Effect of NaCl on dry weight (DW), Na^+^ and K^+^ contents of *C. tinctorius* L. of the whole plant and individual organs after 25 days of treatment in the absence (NaCl, 0 mM) or presence of NaCl (50 mM). Means of eight replicates ± confidence intervals at *p* = 0.05. Means sharing a same letter are not significantly different at *p* = 0.01 (analysis of variance and mean comparison using the Newman–Keuls test).

Examination of Figure 2 showed a modest reduction in whole-plant dry biomass compared with the control. Stems appear to be the organs most affected by salt. Similarly, salt induced reductions in root and leaf dry weights, which were 25 and 14%, respectively, compared to the control.

Cultivation in the presence of NaCl led to a significant accumulation of Na^+^ in the different organs. However, the accumulation levels, reached in roots and leaves after 25 days of treatment, were still higher than those in stems (Figure 2).

As for potassium nutrition, K^+^ levels were maintained at comparable levels in the different organs of plants grown in the absence of salt. The presence of 50 mM NaCl in the culture medium resulted in a disruption of potassium nutrition, especially in roots and leaves (Figure 2).

### 3.2. Effects of NaCl on Essential Oils

#### 3.2.1. Essential Oils Content (µg·g^−1^ DW) of Organs

**Figure 3:**
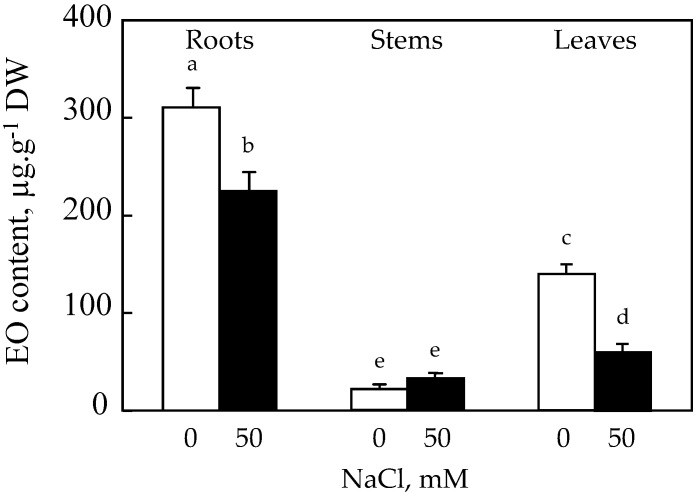
Effect of salinity on EH content (µg·g^−1^ DW) of *C. tinctorius* L. roots, stems, and leaves after 25 days of treatment in the absence (NaCl, 0 mM) or presence of NaCl (50 mM). Means of four replicates ± confidence intervals at *p* = 0.05. Means sharing a same letter are not significantly different at *p* = 0.01 (analysis of variance and mean comparison using the Newman–Keuls test).

In general, the content of EO differs from one organ to another within the same plant; it is in this context that we proceeded to the extraction of EO from roots, stems, and roots (Figure 3).

It appears that the roots of safflower are the most productive organs of EO compared to the leaves and stems. The effect of salt resulted in an inhibition of the EO production capacity of roots by about 28%. Changes in EO content of stems under the effect of 50 mM NaCl showed a significant increase of 35% compared to the control. On the other hand, a decrease of about 60% compared to the control is recorded in the leaves (Figure 3).

#### 3.2.2. Essential Oils Composition (%) of Organs

Capillary gas chromatography analysis of the volatile fraction extracted by hydro-distillation from safflower roots, stems, and leaves grown on control medium identified 34 compounds represented in the chromatograms in Figures S1 and S2.

The EO of roots consists of four main compounds, which are γ-cadinene, β-caryophyllene, β-thujene, and 1-pentadecene, with levels in the order of 17% for the first, 8% for the second, and 7% for the last two compounds. Other compounds are also present at levels of about 2%, such as bornyl acetate and caryophyllene oxide (Table 1).

γ-cadinene > β-caryophyllene > β-thujene > 1-pentadecene > bornyl acetate > caryophyllene oxide >…

The EO of stems is essentially made up of three major compounds, of which 1-pentadecene is the major chemotype, with a rate of 26%; followed by linalool; and myrtenal with a rate of about 5% each. We also note the presence of other relatively important compounds such as methyl-eugenol, trans-α-bergamotene, Z-3-hexenol, elimicin, β-thujene, with a rate of about 2% each (Table 2).

1-pentadecene > linalool > myrtenal > methyl- eugenol > trans-α-bergamotene > …

The EO of leaves is composed essentially of four main compounds, which are β-caryophyllene, 1-pentadecene, terpinolene, and α-terpineol, with levels of about 15.86, 12.29, 11.87, and 6.33% respectively. In addition to these compounds, there are other relatively important ones, such as methyl eugenol (5.56%) and caryophyllene oxide (4.06%) (Table 3).

β-caryophyllene > 1-pentadecene > terpinolene > *α*-terpineol > methyl-eugenol > caryophyllene oxide > …

The presence of NaCl, 50 mM did not generate biosynthesis of new volatile compounds in the roots. However, according to the results in Table 1 and Figure S3, salt caused variations in the EO composition of these organs as shown by the percentages of its different constituents. The salinity caused a very strong decrease of the major chemotype (γ-cadinene) of about 83% in comparison, with an increase of 1-pentadecene of 64% and β-thujene of about 2.5 times compared to the control. There was also a 25% increase in β-caryophyllene. Other lesser compounds also showed variations, namely a reduction of about 8% in bornyl acetate and caryophyllene oxide (Table 1).

β-thujene > 1-pentadecene > β-caryophyllene > γ-cadinene > Z-3-hexenol > …

For the stems, the salt caused changes in the composition of EO. These changes affected the level of 1-pentadecene, which underwent a 30% decrease compared to the control, myrtenal, the level of which increased from 4.75% to trace levels, and linalool, the level of which remained unchanged (Table 2). For the other relatively important compounds, there was an increase of 15, 27, and 72% compared to the control for methyl eugenol, Trans-α-bergamoteme, and Z-3-hexenol, respectively, associated with a 50% decrease in that of β-thujene. As for elemicine, it passed in trace amounts. The shift of murcene (which becomes the second major compound after 1-pentadicene) and α-terpinene from trace levels to 15% and 5% of the EO composition of stems treated with 50 mM NaCl, respectively, was also noted (Table 2).

1-pentadecene > myrcene > linalool > α-terpinene > Z-3-hexenol > methyl eugenol >…

As for the photosynthetic organs, examination of Figure S4 shows that salt treatment did not result in biosynthesis of new volatile compounds. However, the results in Table 3 reveal that the EO composition is altered in response to salt as evidenced by the NaCl-responsive changes in the percentages of the various EO constituents. In fact, with the exception of α-terpineol (which increases by 15% compared to the control), in the presence of salt, we notice a decrease in the level of the other main compounds by 60, 10, and 25% compared to the control, for β-caryophyllene, 1-pentadecene, and terpinolene respectively. For the other relatively important compounds, there is a more than twofold increase compared to the control in the level of caryophyllene oxide, and a slight decrease in that of methyl eugenol. In addition, 1-pentadecene becomes the major chemotype (Table 3).

1-pentadecene > caryophyllene oxide > terpinolene > α-terpineol > β-caryophyllene > …

### 3.3. Antioxidant Activities of Safflower EOs

When the Diphenylpicrylhydrazyl radical (DPPH) solution was mixed with a substance capable of donating a hydrogen atom or an electron, this gave rise to the reduced form of the molecule: DPPH-H with a loss of the purple coloration. Therefore, the greater the antioxidant powers of an essential oil, the less intense the purple coloration. Results of this test read at 517 nm are shown in Table 1. The free radical scavenging activity (ability to remove the DPPH radical) was assessed by determining the concentration corresponding to 50% inhibition (IC_50_). The lowest IC_50_ value corresponds to the highest antiradical activity (Table 4).

The analysis of this table shows that antiradical activity of safflower oil, both in the extracts of control and NaCl-treated plants, was low compared to a positive control (BHT). In the control medium, this activity was relatively higher in leaves than in stems and roots (because leaves have the lowest IC_50_, 167,000 ppm, and therefore the highest antiradical activity). In the presence of NaCl in the culture medium, this activity was decreased until it reaches almost the same level in the different organs (IC_50_ was 280,000 to 312,000 ppm).

The β-carotene bleaching test was used to determine the antioxidant power of the EO studied: the more effective this power, the less the β-carotene discoloration under the action of EO. The slight decrease in absorbance of beta-carotene in the presence of essential oil indicates the effectiveness of the antioxidant power of the latter. Results of this test are shown in Table 5.

Analysis of these results shows that safflower EO has an average antioxidant power in the absence of NaCl. Antioxidant power was similar in leaves and roots and very low in stems. Salt treatment had no effect on the activity of leaves and roots, but it increased the activity of stems. However, this potency was still low compared to the positive control (BHT) and independent of salt treatment.

The total antioxidant capacity of safflower oil shown in Table 6 was low compared to a positive control (Asc), but was higher in the presence than in the absence of NaCl, with the exception of roots. At high EO concentration (50,000 ppm), total antioxidant capacity was higher in the roots of control plants (NaCl, 0 mM) and decreased with NaCl, 50 mM.

In Table 7, results show that the iron-reducing power of safflower oil was low compared to a positive control (Asc). Treatment of plants with 50 mM NaCl had no effect on this antioxidant activity in leaves and stems, while it stimulated it in roots, but never reached the level of the positive control (Asc).

### 3.4. Antifungal Activities of Safflower EOs

**Figure 4:**
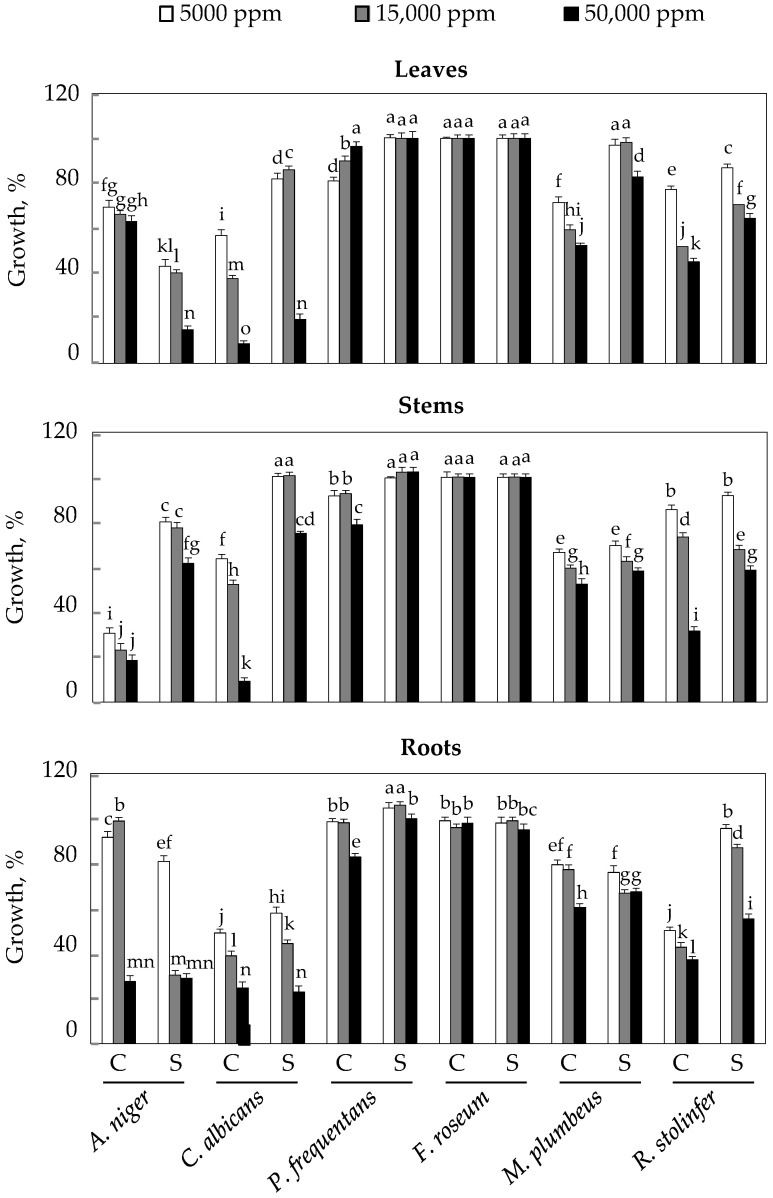
Antifungal activity of EOs extracted from the organs of *C. tinctorius* L. plants grown in the absence (NaCl, 0 mM) or presence of NaCl (50 mM). Means of four replicates ± confidence intervals at *p* = 0.05. Means sharing a same letter are not significantly different at *p* = 0.01 (analysis of variance and mean comparison using the Newman–Keuls test).

The evaluation of the antifungal activity of safflower EO extracted from the leaves, stems, and roots of *C. tinctorius* L. was demonstrated by means of a microdilution test on 96-well plates. Results of this activity are shown in Figure 4. Analysis of this table shows us a significant antifungal activity level of safflower essential oils against *Aspergillus niger*, in the presence of salt, at the level of leaves; and in the absence of salt, at the level of stems; where the percentages of inhibition were 85 and 82% respectively. At the root level, this inhibition was 70% in the absence or presence of NaCl for the concentration of EO (15,000 ppm).

In the case of *Candida albicans*, especially in control plants and considering the concentrations of EOs, the inhibition was about 90% in leaves and stems, while it was 50% in roots.

In other strains, there was a relatively high inhibition against *Mucor plumbeus*—in the absence of salt and on the leaves, and in the absence or presence of NaCl on the stems and roots (inhibition of the order of 40 to 50%). There was also relatively high inhibition against *Rhizopus stolonifer*, at the level of the various organs (inhibition of about 65%), especially in the absence of NaCl.

*Penicillium frequentans* and *Fusarium roseum* showed almost total insensitivity to safflower EO at the level of the different organs.

### 3.5. Antibacterial Activities of Safflower EOs

**Figure 5:**
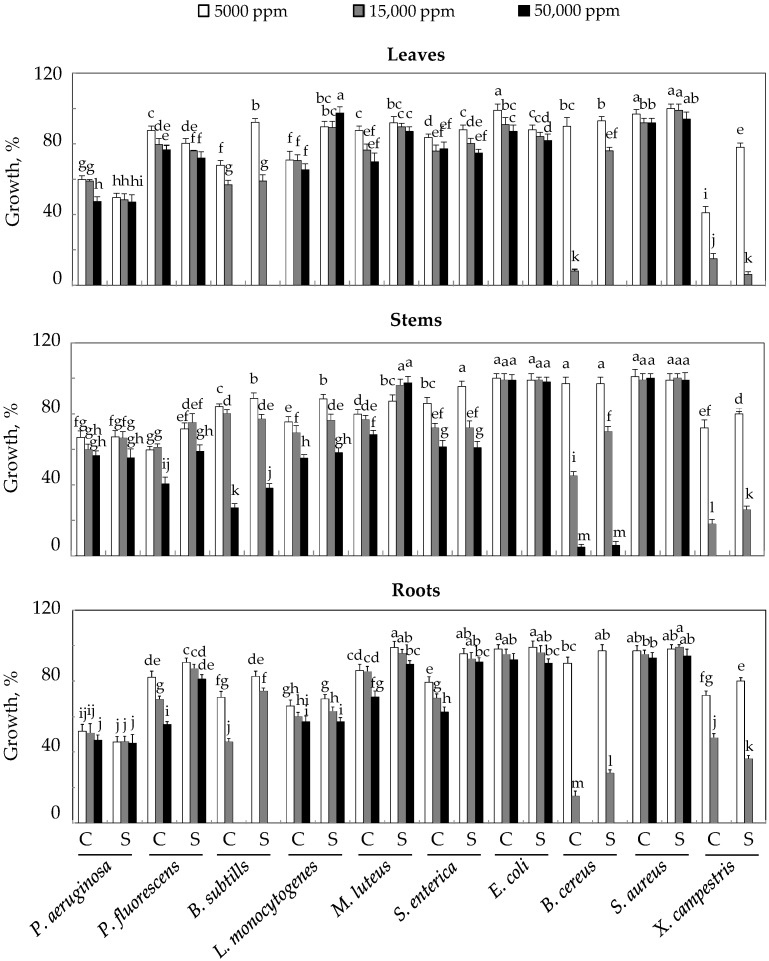
Antibacterial activity of EOs extracted from the organs of *C. tinctorius* L. plants grown in the absence (NaCl, 0 mM) or presence of NaCl (50 mM). Means of four replicates ± confidence intervals at *p* = 0.05. Means sharing a same letter are not significantly different at *p* = 0.01 (analysis of variance and mean comparison using the Newman–Keuls test).

The evaluation of the antibacterial activity of safflower EO extracted from the leaves, stems, and roots of *C. tinctorius* L. was demonstrated by means of a microdilution test on 96-well plates. The microorganisms studied were those considered harmful in the field of cosmetics. Figure 5 summarizes the EO effect of different organs on the growth of bacterial strains.

According to the results in this figure, essential oils of safflower appearto have significant antimicrobial activity against *Bacillus subtilis*, *Bacillus cereus*, and *Xanthomonas campestris* in both control and NaCl-treated plants; for these three strains, a total inhibition of their growth was noted for a concentration of 50,000 ppm EO in leaves and roots, whereas for stems, inhibition was total only for the third strain (*Xanthomonas campestris*). For other strains, this inhibition was variable; it does not exceed 12% for *Escherichia coli* and *Staphylococcus aureus*. In other strains, such as *Pseudomonas aeruginosa*, *Pseudomonas fluorescens*, *Listeria monocytogenes*, *Micrococcus luteus*, and *Salmonella enterica*, inhibition was relatively high (30–50%) for EO, 50,000 ppm. In general, there were no significant differences between control and salt treatment, except for *Listeria monocytogenes* (in leaves), *Pseudomonas fluorescens*, and *Salmonella enterica* ssp stolonifer (in roots), where salinity at 50 mM NaCl decreased the inhibitory effect of EOs in these organs against these strains by about 35%.

## 4. Discussion

Since biomass production is the most important factor determining plant growth, we evaluated the impact of salt treatment on the response of *Carthamus tinctorius* plants. It appears that *C. tinctorius* behaves as a moderately tolerant glycophyte, being able to tolerate a concentration of about 50 mM NaCl. Similar to our results, previous studies have shown that safflower plants affected by salinity tend to be shorter and produce thinner stems [10,32]. The reduction of safflower growth under stress conditions was also reported by Bassil and Kafka [12]. They found a reduction in seedling height of different Canadian varieties. In our case, there was a small reduction in growth. This could be explained either by the fact that the applied salt stress (50 mM NaCl) is not severe enough, or by the maintenance of a normal potassium supply especially at the stem level. The results of the study recommended here are coherent with those of a recent study in which a single safflower genotype was tested in hydroponics using 0, 50, 100, and 150 mM NaCl treatments and showed a significant increase in Na^+^ and a reduction in K^+^/Na^+^ ratio under 150 mM NaCl [33]. The capacity of plants to maintain low Na^+^ concentration in the stem by exclusion or sequestration is identified as a major objective in efforts to improve salinity tolerance in a number of crops [34]. Safflower seems to have strong osmotic tolerance mechanisms when grown in conditions with less than 100 mM Na^+^, being capable of synthesizing a compatible range of solutes and secondary metabolites for osmotic adjustments and water potential preservation [33,35]. At increased salinity levels, safflower appears to be based on ionic exclusion and tissue tolerance mechanisms. Safflower roots have been documented to sequester high levels of Na^+^ and Cl^−^, which suggests that safflower is capable of segregating toxic ions from sensitive organ tissue [14].

The induction of secondary metabolism is one of the characteristics of environmental stresses [36]. Essential oils are biosynthesized secondary metabolite compounds in several species. Our study showed that in response to salt, the essential oil content of safflower in the leaves and roots is decreased but comparable to the control (without NaCl) in the stems. Yield is a major indicator of whether an EO can be carried out on a large scale. Wu et al. [37] showed that the yield of EO in Kushui rose was significantly affected by salt treatment; the yield of EO was enhanced after treatment with salt. This may be due to the fact that NaCl has an elevated osmotic pressure; thus, as plasmolysis is produced in the cell of plant, the yield is enhanced [37]. These results are not in agreement with those found with Sarmoum et al. [22]. Indeed, these authors showed that the yield of essential oil in *Rosmarinus officinalis* was decreased for plants subjected to a salt water regime. Petropoulos et al. [38] reported that to increase the EO yield of plants grown in saline soil, it is important to increase the plant density.

Our results also showed changes in the contents of compounds in safflower EOs, which resulted in a decrease of one of the main compounds (γ-cadinene in roots, myrtenal in stems and β-caryophyllene in leaves) in favor of an increase of the other compound (β-thujone and 1-pentadecene, 1-pentadecene, caryophyllene oxide in roots, stems, and leaves, respectively) under saline conditions. We suspect that the reduction in the content of these compounds in the various organs of salt-treated plants can be the result of a reduction in cell hydration induced by NaCl stress. Khalid and da Silva [24] report that *Calendula officinalis* L. plant exposed to different levels of saline irrigation water composed of NaCl, CaCl_2_, and MgCl_2_ salts enhanced the content of EO and its principal compounds (α-cadinol, γ- and δ-cadinene). Farsaraei et al. [39] reported that basil EO had various responses to salt stress. With the degree of NaCl stress, some of the main compounds such as 1,8-cineole and tau-muurolol increased, while linalool and α-cadinol reduced.

Our results showed that, according to the emission profiles of root, stem, and leaf samples of plants grown without NaCl, the dominant chemical class of EOs in *C. tinctorius* is that of sesquiterpene hydrocarbons (about 35% (data not shown)) such as β-caryophyllene, 1-pentadecene, β-thujone, and terpinolene. Under saline conditions, the total amount of sesquiterpene hydrocarbons varied from 23.22 to 31.84% (data not shown). This shows that this class of EOs was significantly decreased in the presence of 50 mM NaCl. Hendawy et al. [40] showed the opposite in chamomile flowers. Indeed, these authors showed that salinity had no negative impact on yield or essential oil content. The highest yield and essential oil content were obtained under even high salinity conditions and sesquiterpene hydrocarbons were more abundant under these salinity conditions. These authors encourage farmers to grow chamomile where saline soils and/or saline irrigation water are present Hendawy et al. [40]. The difference between our results and those of Hendawy et al. [40] can be explained by the strong importance of the developmental stage of the plants when harvesting and extracting the essential oils. The results presented in our study on safflower were derived from plants in the vegetative stage (early stage).

It could be argued that the essential oil production and accumulation was depending directly on the proper growth and maturation of the oil-producing plants [41]. The reduction in oil production could be due to the reduction in anabolism of the plants. The enhanced oil content of some salt-stressed plants could be explained by the decrease in primary metabolites as a result of the impact of salinity, which makes intermediates available for the synthesis of secondary metabolites. In fact, the impact of salinity on the essential oil and its compounds can be due to its impact on enzymatic activity and metabolism.

The results of antioxidant and antimicrobial activity of essential oils (EOs) extracted from safflower plants grown in the absence and presence of NaCl, 50 mM, revealed that these EOs possess more or less important activities depending on the organ and the nature of the activity [42]. The antioxidant impact of the essential oil of *Carthamus tinctorius* was less than that of the standard antioxidant. The DPPH-scavenging capacity of this oil can be explained by the presence of sesquiterpene hydrocarbons as the main class (35%) in its chemical composition. Khelifa et al. [43] showed that *Ocimum basilicum* EO has significant antioxidant activity (DPPH radical scavenging, and β-carotene bleaching).

After salt treatment, the IC_50_ value of EOs was significantly reduced in roots. This indicates that the ability of EOs extracted from roots to inhibit DPPH radicals is significantly higher than those from the aerial part and that salt treatment has a significant impact on the antioxidant capacity of EOs. These results were in agreement with those found by Ghassemi-Golezani and Farhadi [44] in EO of pennyroyal plants, and by Wu et al. [37] in EOs of salt-treated and non-salt-treated Kushui rose. The antioxidant capacity was higher in EO of salt-treated Kushui rose, which may be associated with the fact that the petal cells expanded and more components were solubilized after salt [37].

In our study, the β-Thujone content of safflower EOs from the roots of salt-treated plants was much higher than that of the control plants, and this is the main reason why the antioxidant capacity of EOs from the roots of salt-treated plants is higher than that of the control plants. 1-pentadecene and β-caryophyllene present in the EOs of the stressed roots could also be responsible for the antioxidant activity with some synergy of minor or major components present. This production of monoterpenes is probably caused by the stimulation of a metabolic pathway that generates terpenoids and therefore monoterpenes, that have an important role in the defense of plants against abiotic stress [45]. Monoterpenes are known to be essential in the response of plants to stress conditions. Terpenoid biosynthetic patterns are mainly influenced by the assimilation of photosynthetic carbon and partially controlled by stress conditions [46]. There are also a number of studies on the antistress activity of monoterpenes in plants under environmental stress [46].

Bornyl acetate, another compound identified in safflower, is considered an antimicrobial (Gr^+^ and Gr^−^). Safflower EO also contains a compound, trans-trans-3,11-tridecadiene-5,7,9-triyne-1,2-diol, which appears to have antifungal properties [19]. *Thymus capitatus* essential oils showed interesting antibacterial activity that was stronger on Gram-positive bacteria, the high activity was found against *Bacillus cereus* [47]. The differences in diffusion of essential oils were caused by the nature of the bacterial cell wall. This clarifies that Gram-positive bacteria were more sensitive to essential oils than Gram-negative bacteria [47].

## 5. Conclusions

The effects of salt on *Carthamus tinctorius* were manifested by a reduction in growth due to a disturbance in potassium nutrition in the roots and leaves.

The EO yield of *C. tinctorius* L was higher in the roots than in the leaves and stems. Salinity caused a decrease in EO yield in the different organs, except for the roots, which showed a 50% increase in yield compared to the control. The compounds identified in the EO of *C. tinctorius* belong to seven chemical classes of which the dominant class is that of sesquiterpenic hydrocarbons and the chemotype is variable according to the organ and the treatment.

Safflower EOs exhibited low antioxidant (total capacity and inhibition of beta-carotene bleaching), antiradical (DPPH radical scavenging), and iron-reducing activities. For antimicrobial activity, only the bacterial strains *Bacillus subtilis*, *Bacillus cereus* and *Xanthomonas campestris* were found to be very sensitive to these oils, since they showed almost total inhibition of their growth.

Safflower EOs showed significant antifungal activity against *Aspergillus niger* and *Candida albicans*. For the other strains, this activity was less important and variable from one strain to another. Salt was found to have no effect on the activities of safflower EOs. The presence of NaCl in *C. tinctorius* plants influenced the antimicrobial activity of EO by increases and decreases in the growth of some strains depending on the organ and the concentration of EO. Only *F. roseum*, *S. aureus*, *L. monocytogenes* (root EO) and *E. coli* (root and stem EOs) did not show a change in growth.

## Figures and Tables

**Table 1:** Effects of NaCl on root essential oil composition (%) of *C. tinctorius* L. Means of four replicates ± confidence intervals at *p* = 0.05. Stars (*) indicate statistically different values compared to the control (Student test with *p* = 0.05). KI: Kovàts index.

Components	KI	NaCl, 0 mM	NaCl, 50 mM
α-pinene	1032	0.04 ± 0.01	0.08 ± 0.02 *
α-thujene	1035	0.05 ± 0.01	0 *
camphene	1076	0.03 ± 0.02	0.05 ± 0.01 *
β-pinene	1118	0.25 ± 0.05	0.19 ± 0.05
sabinene	1132	0.10 ± 0.02	0.09 ± 0.02
δ-3-carene	1159	0.08 ± 0.03	0 *
myrcene	1174	0.56 ± 0.10	0.65 ± 0.20
α-terpinene	1188	0.40 ± 0.10	0.08 ± 0.02 *
myrtenal	1192	1.14 ± 0.20	1.38 ± 0.50
limonene	1203	0.54 ± 0.20	0.22 ± 0.10 *
1.8-cineole	1193	0.10 ± 0.03	0 *
E-2-hexenal	1232	0	0
γ-terpinene	1255	0.38 ± 0.10	0.13 ± 0.02 *
β-ocimene	1266	0	0
P-cymene	1280	0	0
terpinolene	1290	1.26 ± 0.30	0.75 ± 0.03 *
Z-3-hexenol	1370	1.85 ± 0.40	2.27 ± 0.60
β-thujone	1451	6.97 ± 2.10	15.54 ± 2.3 *
trans-α-bergamotene	1438	0.66 ± 0.20	1.20 ± 0.30 *
β-muurolene	1467	1.34 ± 0.50	1.62 ± 0.40
linalool	1553	1.79 ± 0.60	1.99 ± 0.60
bornyl acetate	1597	2.06 ± 0.80	1.75 ± 0.40
terpinene-4-ol	1611	0.55 ± 0.20	0.72 ± 0.20
β-caryophyllene	1612	8.01 ± 2.30	10.65 ± 2.4
α-terpineol	1709	0.42 ± 0.10	0.49 ± 0.40
δ-cadinene	1773	0.53 ± 0.20	0.66 ± 0.20
γ-cadinene	1776	17.29 ± 3.10	2.96 ± 0.50 *
1-pentadecene	1829	6.70 ± 1.50	11.01 ± 3.2 *
methyl-eugenol	2028	1.28 ± 0.50	1.59 ± 0.60
caryophyllene oxide	2008	1.96 ± 0.60	1.83 ± 0.50
spathulenol	2153	0.06 ± 0.03	0.03 ± 0.01 *
cinnamyl acetate	2155	0.14 ± 0.09	0.17 ± 0.05
eugenol	2192	0.58 ± 0.20	0.46 ± 0.10
elemicine	2229	0.38 ± 0.10	0.07 ± 0.01 *

**Table 2:** Effects of NaCl on stem essential oil composition (%) of *C. tinctorius* L. Means of four replicates ± confidence intervals at *p* = 0.05. Stars (*) indicate statistically different values compared to the control (Student test with *p* = 0.05). KI: Kovàts index.

Components	KI	NaCl, 0 mM	NaCl, 50 mM
α-pinene	1032	0.37 ± 0.04	0.34 ± 0.10
α-thujene	1035	tr	tr
camphene	1076	0.04 ± 0.01	0.06 ± 0.01 *
β-pinene	1118	1.24 ± 0.30	0.94 ± 0.20
sabinene	1132	0.09 ± 0.03	0.08 ± 0.03
δ-3-carene	1159	0.06 ± 0.01	0.03 ± 0.01 *
myrcene	1174	0.19 ± 0.04	15.11 ± 2.3 *
α-terpinene	1188	0.20 ± 0.02	4.86 ± 1.10 *
myrtenal	1192	4.79 ± 0.80	tr *
limonene	1203	0.17 ± 0.02	0.22 ± 0.10
1.8-cineole	1193	0.24 ± 0.10	0.06 ± 0.02 *
E-2-hexenal	1232	tr	tr
γ-terpinene	1255	0.25 ± 0. 10	0.14 ± 0.10
β-ocimene	1266	0.35 ± 0.10	0.32 ± 0.10
P-cymene	1280	0.18 ± 0.01	0.15 ± 0.05
terpinolene	1290	0.59 ± 0.20	0.45 ± 0.20
Z-3-hexenol	1370	2.06 ± 0.60	3.55 ± 0.90 *
β-thujone	1451	2.16 ± 0.80	1.18 ± 0.50 *
trans-α-bergamotene	1438	2.12 ± 0.50	2.70 ± 0.50
β-muurolene	1467	0.75 ± 0.20	0.60 ± 0.10
linalool	1553	4.87 ± 1.40	4.98 ± 1.30
bornyl acetate	1597	0.70 ± 0.10	0.75 ± 0.20
terpinene-4-ol	1611	1.98 ± 0.70	1.09 ± 0.60 *
β-caryophyllene	1612	1.62 ± 0.80	0.73 ± 0.30 *
α-terpineol	1709	1.37 ± 0.20	1.56 ± 0.20
δ-cadinene	1773	0.50 ± 0.02	0.74 ± 0.10 *
γ-cadinene	1776	0.26 ± 0.04	0.22 ± 0.10
1-pentadecene	1829	26.32 ± 4.1	18.24 ± 3.1 *
methyl-eugenol	2028	2.86 ± 0.30	3.31 ± 1.10
caryophyllene oxide	2008	0.58 ± 0.15	0.58 ± 0.20
spathulenol	2153	0.18 ± 0.04	0.22 ± 0.05
cinnamyl acetate	2155	0.32 ± 0.10	0.53 ± 0.10 *
eugenol	2192	0.88 ± 0.40	0.89 ± 0.30
elemicine	2229	2.10 ± 0.50	0.17 ± 0.03 *

**Table 3:** Effects of NaCl on leaf essential oil composition (%) of *C. tinctorius* L. Means of four replicates ± confidence intervals at *p* = 0.05. Stars (*) indicate statistically different values compared to the control (Student test with *p* = 0.05). KI: Kovàts index.

Components	KI	NaCl, 0 mM	NaCl, 50 mM
α-pinene	1032	0.95 ± 0.20	1.05 ± 0.30
α-thujene	1035	0.72 ± 0.10	0.79 ± 0.30
camphene	1076	1.58 ± 0.30	2.09 ± 0.60
β-pinene	1118	0.68 ± 0.20	1.32 ± 0.20 *
sabinene	1132	0.30 ± 0.10	0.44 ± 0.10 *
δ-3-carene	1159	0.43 ± 0.10	0.23 ± 0.10 *
myrcene	1174	0.37 ± 0.10	3.11 ± 0.20 *
α-terpinene	1188	tr	0.19 ± 0.10 *
myrtenal	1192	0.44 ± 0.10	0.86 ± 0.20 *
limonene	1203	tr	0.17 ± 0.02 *
1.8-cineole	1193	0.50 ± 0.20	0.33 ± 0.01
E-2-hexenal	1232	tr	0.02 ± 0.01 *
γ-terpinene	1255	0.30 ± 0.10	0.21 ± 0.10
β-ocimene	1266	0.29 ± 0.10	0.06 ± 0.01 *
P-cymene	1280	tr	0.08 ± 0.02 *
terpinolene	1290	11.87 ± 3.2	8.73 ± 0.90 *
Z-3-hexenol	1370	0.27 ± 0.10	0.67 ± 0.20 *
β-thujone	1451	0.05 ± 0	tr *
trans-α-bergamotene	1438	tr	0.17 ± 0.05 *
β-muurolene	1467	0.30 ± 0.10	0.27 ± 0.10
linalool	1553	2.16 ± 0.50	2.76 ± 0.50
bornyl acetate	1597	0.52 ± 0.10	1.26 ± 0.60 *
terpinene-4-ol	1611	0.81 ± 0.20	0.82 ± 0.50 *
β-caryophyllene	1612	15.86 ± 3.5	6.32 ± 2.10 *
α-terpineol	1709	6.33 ± 1.30	7.26 ± 1.60
δ-cadinene	1773	2.66 ± 1.20	3.31 ± 0.50
γ-cadinene	1776	1.64 ± 0.50	1.81 ± 0.30
1-pentadecene	1829	12.29 ± 2.3	11.34 ± 3.1
methyl-eugenol	2028	5.56 ± 1.30	5.49 ± 1.60
caryophyllene oxide	2008	4.06 ± 1.20	8.88 ± 2.10 *
spathulenol	2153	1.60 ± 0.50	1.52 ± 0.50
cinnamyl acetate	2155	1.07 ± 0.40	1.09 ± 0.10
eugenol	2192	1.15 ± 0.20	1.10 ± 0.20
elemicine	2229	1.46 ± 0.60	2.49 ± 0.60 *

**Table 4:** Antiradical activity assessed by DPPH radical scavenging capacity of EOs extracted from the organs of *C. tinctorius* L. plants grown in the absence (NaCl, 0 mM) or presence of NaCl (50 mM). Means of four replicates ± confidence intervals at *p* = 0.05. Means sharing a same letter are not significantly different at *p* = 0.01 (analysis of variance and mean comparison using the Newman–Keuls test). DPPH: Diphenylpicrylhydrazyl radical, EO: Essential oil, IC_50_: the minimum inhibition concentration at 50%, ppm: part per million.

***C. tinctorius***	**IC50 (Leaves) ppm**	**IC50 (Stems) ppm**	**IC50 (Roots) ppm**
EO _NaCl, 0 mM (50,000 ppm)_	167,000 ± 5053 ^a^	192,307 ± 3246 ^b^	312,500 ± 12,701 ^d^
EO _NaCl, 50 mM (50,000 ppm)_	280,000 ± 6643 ^c^	312,000 ± 12,233 ^d^	280,000 ± 9169 ^c^
Positive control _(5000 ppm)_	2874	2874	2874

**Table 5:** Antioxidant activity: inhibition of β-carotene bleaching by EOs extracted from the organs of *C. tinctorius* L. plants grown in the absence (NaCl, 0 mM) or presence of NaCl (50 mM). Means of four replicates ± confidence intervals at *p* = 0.05. Means sharing a same letter are not significantly different at *p* = 0.01 (analysis of variance and mean comparison using the Newman–Keuls test). EO: Essential oil, IC_50_: the minimum inhibition concentration at 50%, ppm: part per million.

*C. tinctorius*	IC50 (Leaves) ppm	IC50 (Stems) ppm	IC50 (Roots) ppm
EO _NaCl, 0 mM (50,000 ppm)_	48,076 ± 343 ^b^	138,889 ± 701 ^d^	49,019 ± 304 ^c^
EO _NaCl, 50 mM (50,000 ppm)_	46,296 ± 252 ^a^	48,079 ± 390 ^b^	47,170 ± 386 ^b^
Positive control _(5000 ppm)_	2874	2874	2874

**Table 6:** Total antioxidant capacity of EOs extracted from the organs of *C. tinctorius* L. plants grown in the absence (NaCl, 0 mM) or presence of NaCl (50 mM). Means of four replicates ± confidence intervals at *p* = 0.05. Means sharing a same letter are not significantly different at *p* = 0.01 (analysis of variance and mean comparison using the Newman–Keuls test). Asc: ascorbic acid, m OD: mean optical density, ppm: part per million.

NaCl, 0 mM	m OD (Leaves)	m OD (Stems)	m OD (Roots)
Concentrations ppm			
5000	0 ± 0 ^i^	0 ± 0 ^i^	0 ± 0 ^i^
15,000	0.007 ± 0.001 ^g^	0 ± 0 ^i^	0.004 ± 0.000 ^h^
50,000	0.050 ± 0.005 ^c^	0.014 ± 0.001 ^f^	0.140 ± 0.008 ^a^
Asc	0.339	0.339	0.339
**NaCl, 0 mM**	**m OD (Leaves)**	**m OD (Stems)**	**m OD (Roots)**
Concentrations ppm			
5000	0 ± 0 ^i^	0.008 ± 0.001 ^g^	0.009 ± 0.001 ^g^
15,000	0.029 ± 0.003 ^d^	0.018 ± 0.002 ^e^	0.050 ± 0.004 ^c^
50,000	0.110 ± 0.011 ^b^	0.051 ± 0.005 ^c^	0.101 ± 0.018 ^b^
Asc	0.339	0.339	0.339

**Table 7:** Iron-reducing capacity of EOs extracted from the organs of *C. tinctorius* L. plants grown in the absence (NaCl, 0 mM) or presence of NaCl (50 mM). Means of four replicates ± confidence intervals at *p* = 0.05. Means sharing a same letter are not significantly different at *p* = 0.01 (analysis of variance and mean comparison with Newman–Keuls test). Asc: ascorbic acid, m OD: mean optical density, ppm: part per million.

NaCl, 0 mM	m OD (Leaves)	m OD (Stems)	m OD (Roots)
Concentrations ppm			
5000	0.028 ± 0.001 ^g^	0.029 ± 0.002 ^g^	0.025 ± 0.002 ^h^
15,000	0.048 ± 0.003 ^de^	0.037 ± 0.004 ^f^	0.025 ± 0.001 ^h^
50,000	0.078 ± 0.002 ^b^	0.051 ± 0.003 ^d^	0.045 ± 0.004 ^e^
Asc	0.339	0.339	0.339
**NaCl, 0 mM**	**m OD (Leaves)**	**m OD (Stems)**	**m OD (Roots)**
Concentrations ppm			
5000	0.027 ± 0.003 ^gh^	0.020 ± 0.002 ^i^	0.045 ± 0.003 ^e^
15,000	0.039 ± 0.002 ^f^	0.026 ± 0.002 ^h^	0.058 ± 0.003 ^c^
50,000	0.078 ± 0.002 ^b^	0.051 ± 0.002 ^d^	0.118 ± 0.005 ^a^
Asc	0.339	0.339	0.339

## Data Availability

Not applicable.

## Supplementary Materials

The following are available online at https://www.mdpi.com/article/10.3390/plants10122809/s1, Figure S1: EO chromatogram of C. tinctorius L. roots from plants grown on control medium, Figure S2: Chromatogram of EO of C. tinctorius L. leaves from plants grown on control medium, Figure S3: Chromatogram of EO of C. tinctorius L. roots from plants treated with NaCl, 50 mM, Figure S4: Chromatogram of EO of C. tinctorius L. leaves from plants treated with NaCl, 50 mM.
