# Supplementary material for: Effects of NaCl on Antioxidant, Antifungal, and Antibacterial Activities in Safflower Essential Oils
Source: Plants (Basel). 2021 Dec 18;10(12):2809. doi: 10.3390/plants10122809 (PMC8706115; doi:10.3390/plants10122809)
Supplement: Supplementary file 1 [file plants-10-02809-s001.zip › plants-1445539-supplementary.pdf]

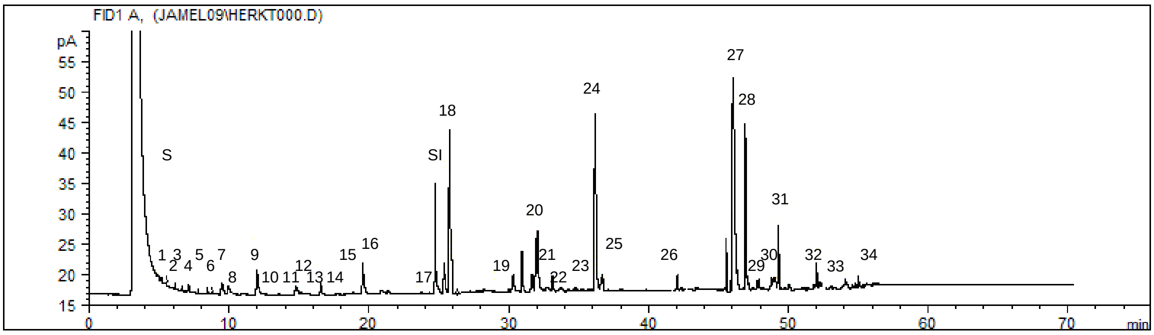

Figure S1. EO chromatogram of *C. tinctorius* L. roots from plants grown on control medium
(s: solvent, SI: internal standard,1: *α*-Pinene, 2: *α*-Thujene, 3: Camphene, 4: *β*-Pinene, 5: Sabinene, 6: *δ*-3 Carene, 7: Myrcene, 8: *α*-Terpinene, 9: Myrtenal, 10: Limonene, 11: 1.8 Cineole, 12: *E*-2-Hexenal, 13: *γ*-Terpinene, 14: *β*-Ocimene, 15: *P*-Cymene, 16: Terpinolene, 17: *Z*-3-Hexenol, 18: *β*-Thujone, 19: *Trans*-*α*-Bergamoteme, 20: *β*-Muurolene, 21: Linalool, 22: Bornyl acetate, 23: Terpinene-4-ol, 24: *β*-Caryophyllene, 25: *α*-Terpineol, 26: δ-Cadinene, 27: *γ*-Cadinene, 28: *1*-pentadicene, 29: Methyl-eugenol, 30: Caryophyllene oxide, 31: Spathulenol, 32: Cinnamyl acetate, 33: Eugenol, 34: Elemicine).

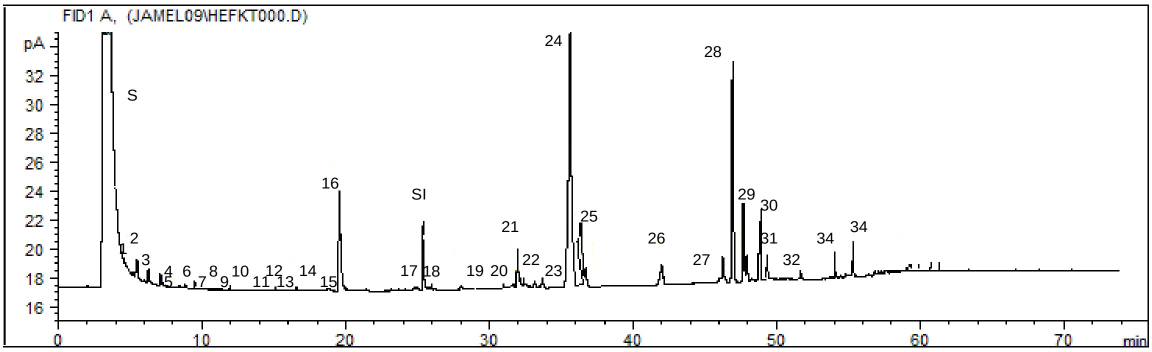

Figure S2. Chromatogram of EO of *C. tinctorius* L. leaves from plants grown on control medium.
(s: solvent, SI: internal standard,1: *α*-Pinene, 2: *α*-Thujene, 3: Camphene, 4: *β*-Pinene, 5: Sabinene, 6: *δ*-3 Carene, 7: Myrcene, 8: *α*-Terpinene, 9: Myrtenal, 10: Limonene, 11: 1.8 Cineole, 12: *E*-2-Hexenal, 13: *γ*-Terpinene, 14: *β*-Ocimene, 15: *P*-Cymene, 16: Terpinolene, 17: *Z*-3-Hexenol, 18: *β*-Thujone, 19: *Trans*-*α*-Bergamoteme, 20: *β*-Muurolene, 21: Linalool, 22: Bornyl acetate, 23: Terpinene-4-ol, 24: *β*-Caryophyllene, 25: *α*-Terpineol, 26: δ-Cadinene, 27: *γ*-Cadinene, 28: *1*-pentadicene, 29: Methyl-eugenol, 30: Caryophyllene oxide, 31: Spathulenol, 32: Cinnamyl acetate, 33: Eugenol, 34: Elemicine).

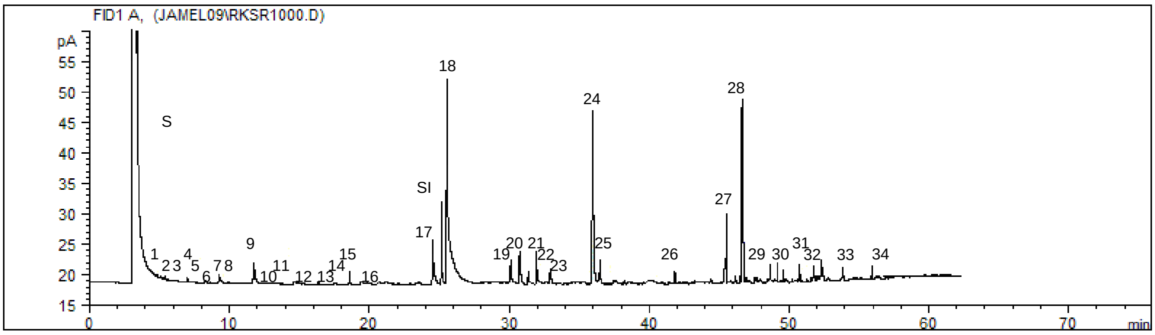

Figure S3. Chromatogram of EO of *C. tinctorius* L. roots from plants treated with NaCl, 50 mM.
(s: solvent, SI: internal standard,1: *α*-Pinene, 2: *α*-Thujene, 3: Camphene, 4: *β*-Pinene, 5: Sabinene, 6: *δ*-3 Carene, 7: Myrcene, 8: *α*-Terpinene, 9: Myrtenal, 10: Limonene, 11: 1.8 Cineole, 12: *E*-2-Hexenal, 13: *γ*-Terpinene, 14: *β*-Ocimene, 15: *P*-Cymene, 16: Terpinolene, 17: *Z*-3-Hexenol, 18: *β*-Thujone, 19: *Trans*-*α*-Bergamoteme, 20: *β*-Muurolene, 21: Linalool, 22: Bornyl acetate, 23: Terpinene-4-ol, 24: *β*-Caryophyllene, 25: *α*-Terpineol, 26: δ-Cadinene, 27: *γ*-Cadinene, 28: *1*-pentadicene, 29: Methyl-eugenol, 30: Caryophyllene oxide, 31: Spathulenol, 32: Cinnamyl acetate, 33: Eugenol, 34: Elemicine).

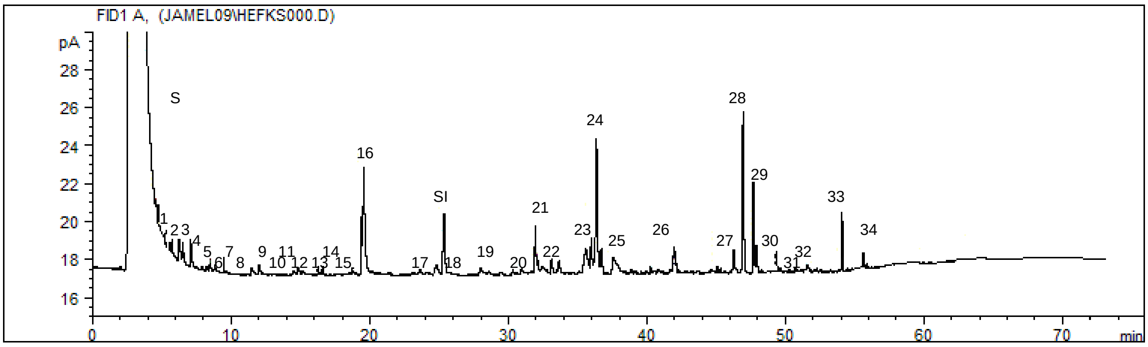

Figure S4. Chromatogram of EO of *C. tinctorius* L. leaves from plants treated with NaCl, 50 mM.
(s: solvent, SI: internal standard,1: α-Pinene, 2: α-Thujene, 3: Camphene, 4: β-Pinene, 5: Sabinene, 6: δ-3 Carene, 7: Myrcene, 8: α-Terpinene, 9: Myrtenal, 10: Limonene, 11: 1.8 Cineole, 12: *E*-2-Hexenal, 13: γ-Terpinene, 14: β-Ocimene, 15: *P*-Cymene, 16: Terpinolene, 17: *Z*-3-Hexenol, 18: β-Thujone, 19: *Trans*-α-Bergamoteme, 20: β-Muurolene, 21: Linalool, 22: Bornyl acetate, 23: Terpinene-4-ol, 24: β-Caryophyllene, 25: α-Terpineol, 26: δ-Cadinene, 27: γ-Cadinene, 28: *1*-pentadicene, 29: Methyl-eugenol, 30: Caryophyllene oxide, 31: Spathulenol, 32: Cinnamyl acetate, 33: Eugenol, 34: Elemicine).
